# Circulating tumor cells promote the metastatic colonization of disseminated carcinoma cells by inducing systemic inflammation

**DOI:** 10.18632/oncotarget.16084

**Published:** 2017-03-10

**Authors:** Yong-Chao Li, Jiu-Ming Zou, Chao Luo, Yu Shu, Jing Luo, Jian Qin, Yu Wang, Dong Li, Shan-Shan Wang, Gang Chi, Fang Guo, Gui-Mei Zhang, Zuo-Hua Feng

**Affiliations:** ^1^ Department of Biochemistry and Molecular Biology, School of Basic Medicine, Tongji Medical College, Huazhong University of Science and Technology, Wuhan 430030, The People's Republic of China

**Keywords:** circulating tumor cells (CTCs), disseminated carcinoma cells, metastatic colonization, inflammation, neutrophils

## Abstract

Circulating tumor cells (CTCs) have been studied well in the prognosis for malignant diseases as liquid biopsy, but their contribution to tumor metastasis is not clearly defined. Here we report that CTCs could promote the metastatic colonization of disseminated carcinoma cells by inducing systemic inflammation and neutrophil recruitment to pre-metastatic organs. Depletion of neutrophils *in vivo* could effectively abrogate the promoting effect of CTCs on tumor cell metastasis. In the presence of CTCs, the pro-tumor function of neutrophils was augmented, whereas the antitumor function of neutrophils was suppressed. Mechanically, CTC-derived ligands for TLR2 and TLR4 (TLR2/4) induced the systemic inflammation, thus increasing the production of proinflammatory cytokines such as G-CSF and IL-6 that could induce the conversion of neutrophil function from tumor-suppressing to tumor-promoting. Moreover, CTCs induced the production of endogenous TLR2/4 ligands such as S100A8, S100A9, and SAA3, which may amplify the stimulating effect that induces the expression of proinflammatory cytokines. The promoting effect of CTCs on tumor cell metastasis could be abrogated by suppressing inflammatory response with IL-37, an anti-inflammatory cytokine, or blocking CTC-derived ligands for TLR2/4. Identification of the metastatic axis of CTCs/systemic inflammation/neutrophils may provide potential targets for preventing tumor cell metastasis.

## INTRODUCTION

Despite considerable advancements in diagnosis and treatment of solid tumors, distant metastases remain the main cause of cancer-related mortality. The development of metastasis is a multi-step process. The successful initiation of metastatic growth, termed “metastatic colonization”, is the final and inefficient step for many types of cancer [1, 2]. The critical determinants of whether the disseminated carcinoma cells (the tumor cells disseminated to a distant organ) could succeed in forming significant metastases include not only the intrinsic properties of cancer cells themselves but also local tissue microenvironment and systemic environment such as the cancer-related inflammation.

During the metastatic process, single or small clusters of tumor cells shed from the primary tumors and passage to distant organs. Tumor cells that spread in the peripheral blood are characterized as circulating tumor cells (CTCs) [3]. CTCs are commonly detectable in cancer patients at varying frequency. In some patients, CTCs could reach higher counts in blood (10^2^/ml~10^3^/ml), resulting in the poorer prognosis and shorter survival of the patients [4–6]. CTCs promote tumor progression via multiple pathways, including modulating tumor microenvironment [7–9]. Accordingly, CTCs might be not only the source of metastatic cells in target organs, but also an important factor for promoting tumor cell metastasis. However, little is known about whether CTCs could influence the metastatic colonization of disseminated carcinoma cells by orchestrating tumor associated-inflammation.

The tumor-promoting effects of inflammation are now widely recognized and better understood [10, 11]. Polymorphonuclear leukocytes (PMNs or neutrophils) are the major part of the infiltrated inflammatory cells found in a wide variety of human cancers and animal models [12]. Neutrophils have been found to promote multiple tumor processes, including tumorigenesis, tumor growth, and metastasis. The role of neutrophils in the formation of lung metastasis has been well demonstrated [13]. Neutrophils could release MMP-9 [14], Bv8 [15], Arginase-1 (Arg-1) [16], and inducible nitric oxide synthase (iNOS) [17], which not only promote tumor angiogenesis [14, 15, 17], but also suppress the proliferation and cytotoxicity of CD8^+^ T cells [16], thus suppressing the anti-tumor immune response and promoting tumor growth and metastasis. Neutrophils can alter their polarization state in the tumor-bearing host, switching from suppressing to promoting roles in tumor metastasis [18, 19]. However, little is known about whether CTCs could induce the conversion of neutrophil function.

In this study, we investigated how CTCs promote tumor metastasis, and found that CTCs could initiate pro-metastatic inflammatory responses, promote neutrophilic infiltration in lung, and induce the conversion of neutrophil function to pro-metastasis, consequently promoting the metastatic colonization of disseminated carcinoma cells.

## RESULTS

### CTCs promote the metastatic colonization of disseminated carcinoma cells

**Figure 1:**
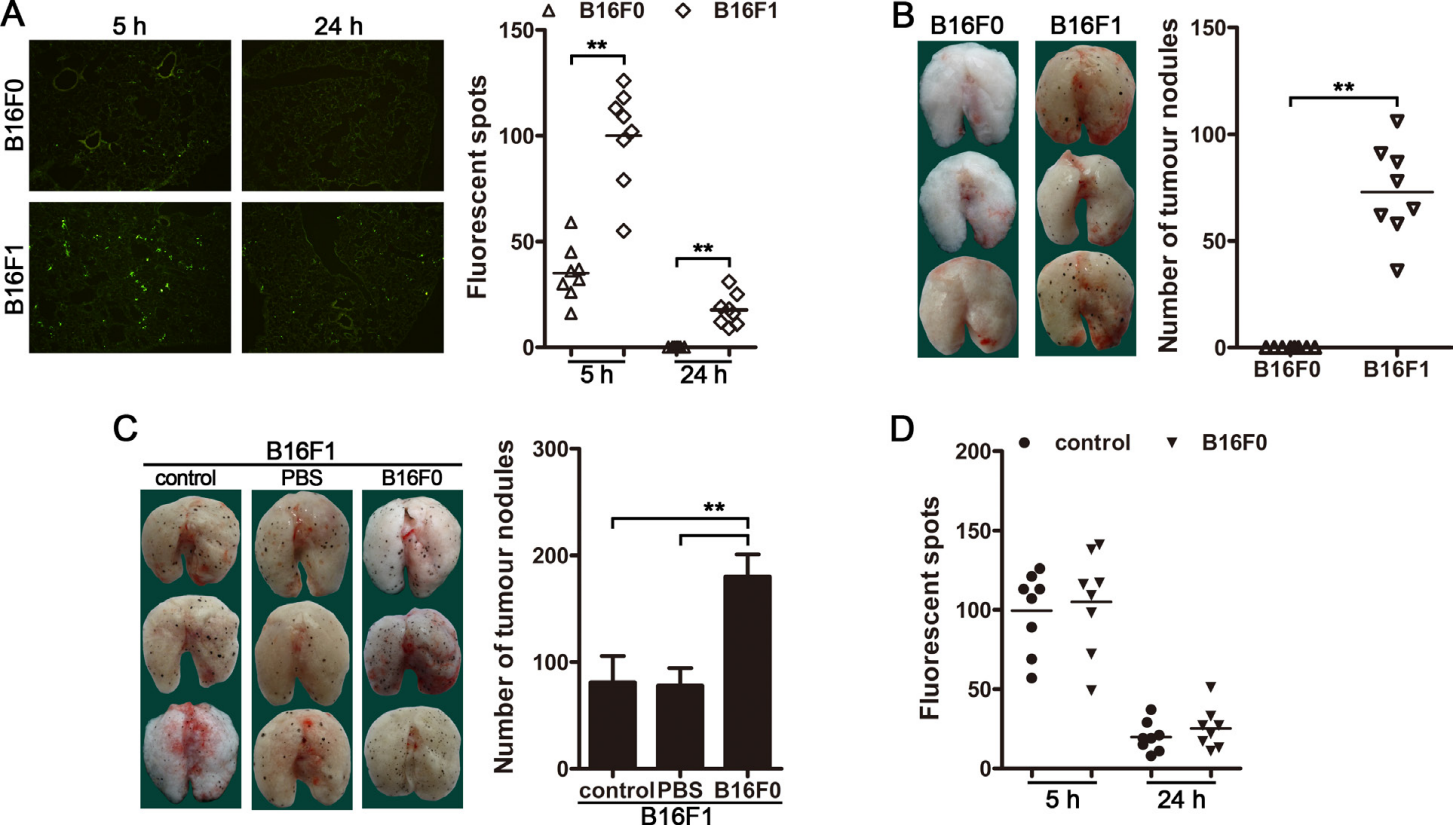
CTCs promote the metastatic colonization of disseminated carcinoma cells (**A**) Mice were injected with CFSE-labeled B16F1 or B1F0 cells (5 × 10^5^ cells/mice) via tail vein. The mice (*n* = 8 in each group) were sacrificed 5 h (for analysis of tumor cell arrest) or 24 h (for analysis of extravasation) after tumor cell injection. Tumor cells in frozen sections were visualized by fluorescence microscopy (left) and counted (right). (**B** and **C**) Mice (*n* = 8 in each group) were intravenously inoculated with B16F1 cells and/or B16F0 cells as described in Methods. Metastatic nodules on the surface of lung (left) were counted (right). PBS was used as control (C). (**D**) Mice were injected with CFSE-labeled B16F1 cells 12 h after non-labeled B16F0 cells injection via tail vein. The mice (*n* = 8 in each group) were sacrificed 5 h or 24 h after B16F1 cell injection. Tumor cells in frozen sections were counted. **p < 0.01.

Recent studies have demonstrated that the vast majority of CTCs are unable to form a metastatic clone [1, 2, 20]. In order to explore whether CTCs could influence the metastatic colonization of the disseminated carcinoma cells, we used B16F0 cells, a non-metastatic melanoma cell line, which could not extravasate (Figure 1A) to form visible metastatic nodules in the lungs (Figure 1B) after intravenous (i.v.) injection. B16F1 cells, a metastatic melanoma cell line, were used to establish the metastasis model. To provide CTCs, B16F0 cells were intravenously injected to the mice inoculated with B16F1 cells. Intriguingly, the intravenously injected B16F0 cells (circulating B16F0 cells) could promote the pulmonary metastasis of intravenously inoculated B16F1 cells (Figure 1C and Supplementary Figure 1A). Furthermore, the intravenous injection of B16F0 cells could not influence the extravasation of B16F1 cells (Figure 1D and Supplementary Figure 1B), indicating that the circulating B16F0 cells may promote the metastatic colonization of the disseminated B16F1 cells and thus contribute to the metastasis. Consistently, the growth of B16F1 cells after intramuscular inoculation could also be promoted by circulating B16F0 cells (Supplementary Figure 1C). Taken together, these results indicated that, in addition to seeding metastases by themselves, CTCs could promote tumor metastasis by facilitating the metastatic colonization of disseminated carcinoma cells.

### Inflammation is involved in the promoting effect of CTCs on metastasis

**Figure 2:**
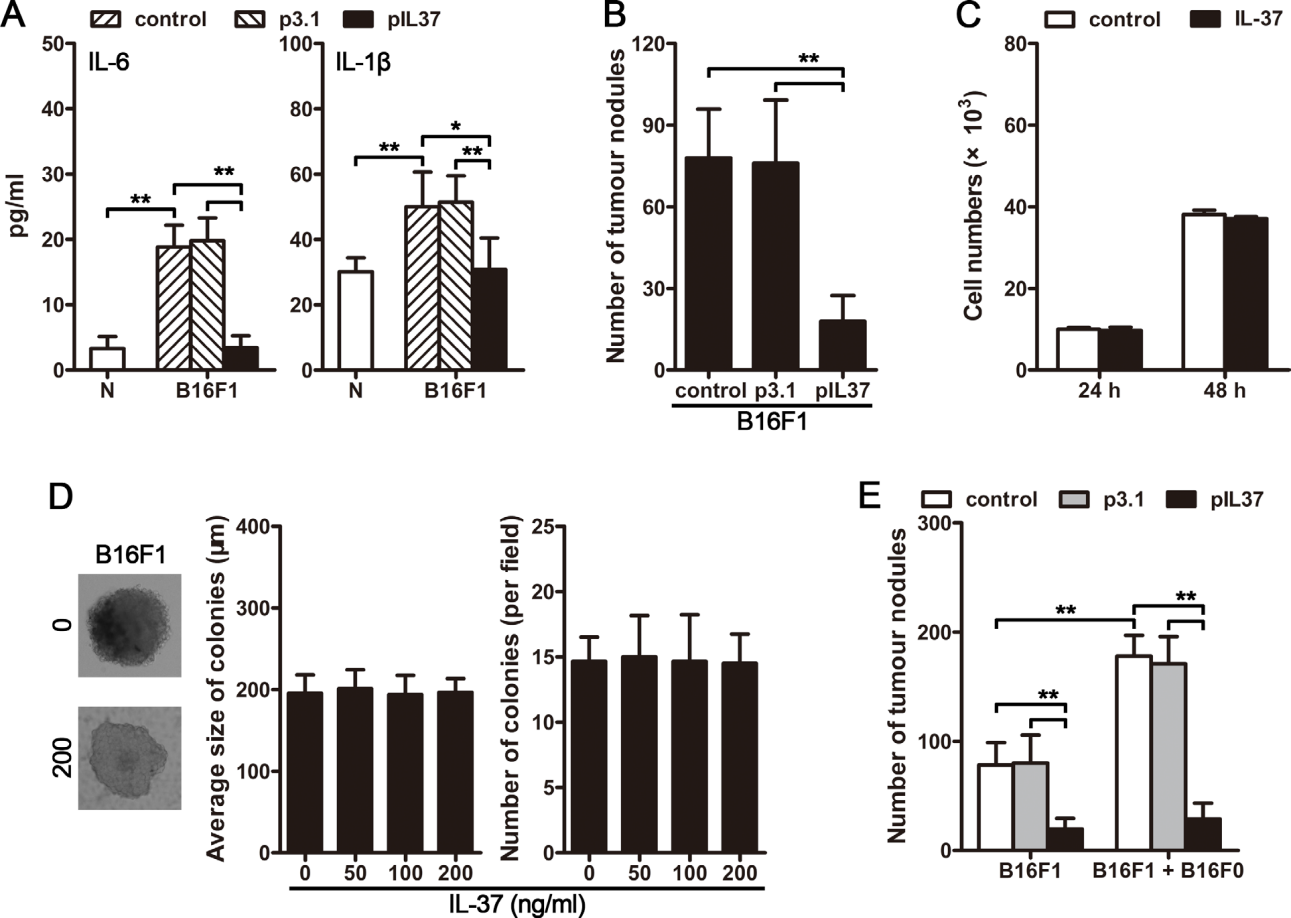
CTC-induced systemic inflammation is essential for the promoting effect of CTCs on tumor metastasis (**A**) Mice were intravenously inoculated with B16F1 cells, and received pIL37 plasmid treatment. Serum levels of IL-6 and IL-1β were detected by ELISA on day 4 after primary inoculation. (**B**) Mice (*n* = 9 in each group) were intravenously inoculated with B16F1 cells, and received the treatment by i.v. injection of pIL37 plasmid. Metastatic nodules on the surface of lung were counted. (**C**) B16F1 cells were cultured in the absence or presence of IL-37 (200 ng/ml) for 24 h or 48 h. Then, CCK-8 cell proliferation assay was performed. (**D**) B16F1 cells were cultured in soft agar for 3 weeks in the absence or presence of IL-37 at the indicated concentration. The representative colonies in the absence (0) or presence of 200 ng/ml IL-37 (200) were photographed (left). The average size of colonies was calculated (middle), and the colonies were counted (right). (**E**) Mice received the i.v. injection of B16F1 cells with or without B16F0 cells. The mice (*n* = 9 in each group) were treated with i.v. injection of pIL37 plasmid. Metastatic nodules on the surface of lung were counted. Data are pooled from three independent experiments with a total of six samples in each group (B, C). *p < 0.05, **p < 0.01.

Recent data have expanded the concept that inflammation, especially the chronic inflammation, is a critical component for promoting tumor growth and metastasis [10, 11, 21]. We next investigated whether inflammation might be involved in the promoting effect of CTCs on metastasis. For this purpose, we expressed IL-37, an anti-inflammatory cytokine [22] that suppresses the expression of multiple pro-inflammatory cytokines and may also has an anti-tumor effect [22–25], in B16F1-inoculated mice. The inoculation of B16F1 cells induced the inflammation *in vivo*, as evidenced by the increase of IL-6 and IL-1β in the serum of mice (Figure 2A). The *in vivo* expression of IL-37 resulted in a significant decrease in lung metastases in B16F1-bearing mice (Figure 2B), accompanied by the inhibition of B16F1 cell-induced inflammation (Figure 2A). To exclude the possibility that IL-37 may have a direct effect on tumor cells, we tested the effect of IL-37 on tumor cell proliferation and colonization. The proliferation *in vitro* and colony-formation in soft agar of B16F1 cells were not influenced by IL-37 (Figure 2C and 2D). Consistently, B16F1 cells did not express the gene of IL-37 receptor, *Sigirr* (*Il1r8*) and *Il18r* (Supplementary Figure 2A). Collectively, these results validated that inflammation is required for tumor metastasis, and that IL-37 could effectively inhibit tumor metastasis by suppressing the tumor-associated inflammatory response.

To further ascertain whether CTCs were involved in inducing systemic inflammation that promotes the metastatic colonization of the disseminated carcinoma cells, we next investigated whether circulating B16F0 cells could induce a systemic inflammatory response. The results showed that, after intravenous inoculation of B16F1 cells, the circulating B16F0 cells could enhance the inflammatory response *in vivo*, as evidenced by the elevated levels of IL-6 and IL-1β in the serum (Supplementary Figure 2B). Moreover, the B16F0-enhanced inflammation could be reversed by *in vivo* expression of IL-37 (Supplementary Figure 2B). Accordingly, the promoting effect of circulating B16F0 cells on the metastatic colonization of disseminated B16F1 cells was also suppressed by IL-37 (Figure 2E). Together, these observations suggested that CTCs could induce systemic inflammation and consequently promoting the metastatic colonization of disseminated carcinoma cells.

### Neutrophils are required for CTCs to promote tumor metastasis

**Figure 3:**
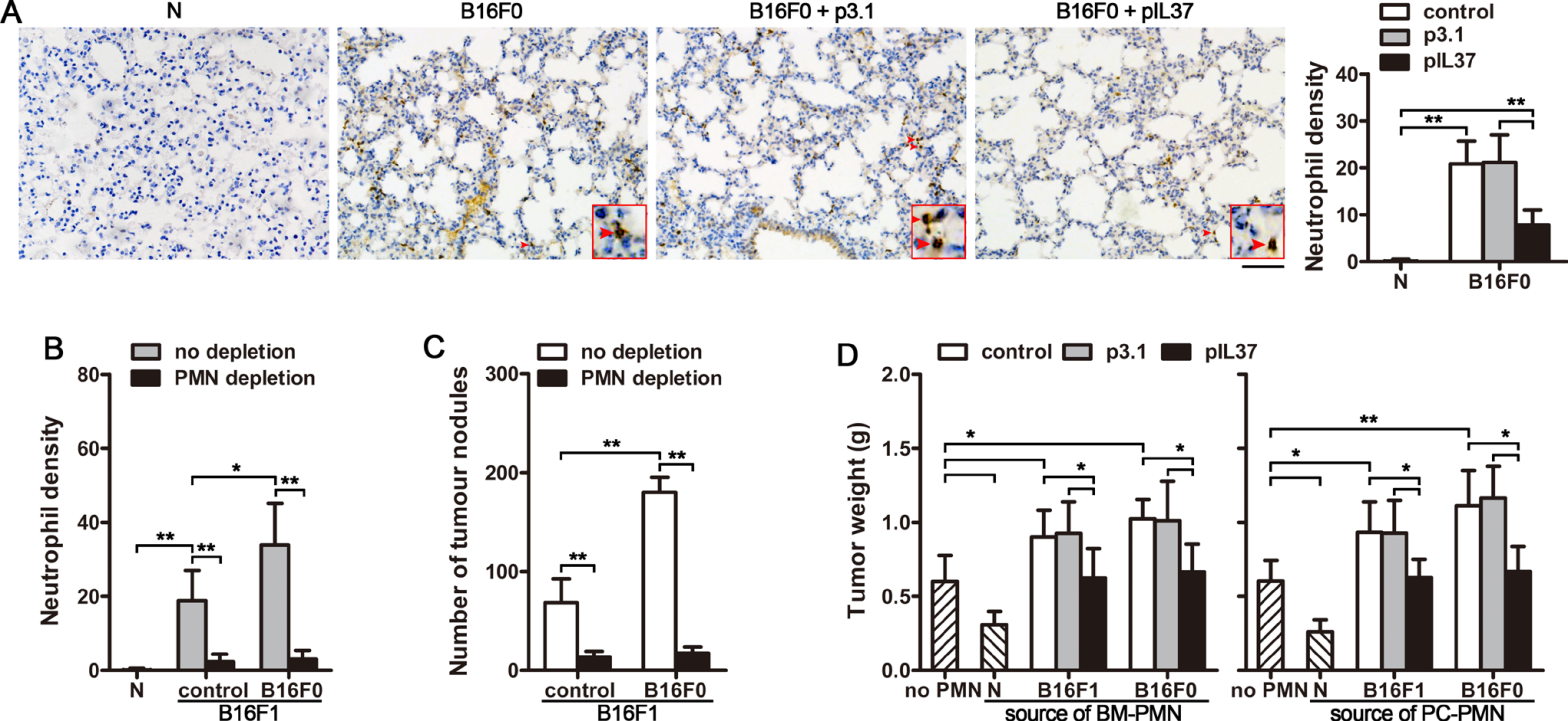
Neutrophils are involved in the pro-metastasis effect of CTCs (**A**) Mice (*n* = 9 in each group) were inoculated with B16F0 cells intravenously, and received pIL37 plasmid treatment. Lung tissue sections were prepared and subjected to immunohistochemical staining to analyze the infiltration of neutrophils on d12 after primary inoculation (left, Bar, 50 μm). Neutrophil density (number of neutrophils per microscopic field) was determined (right). Naive mice (N) were used as control. (**B** and **C**) Mice (*n* = 9 in each group) received the i.v. injection of B16F1 cells with or without B16F0 cells. Neutrophils were depleted *in vivo* as indicated. (B) The infiltrated neutrophils in lung tissue were analyzed. The neutrophil density was determined after immunohistochemical staining. (C) The metastatic nodules on the surface of lung were counted. (**D**) Neutrophils were isolated from the bone marrow (BM) or peritoneal cavity (PC) of naive mice, B16F1-bearing mice, cir-B16F0-mice with or without treatment with i.v. injection of pIL37 plasmid. The neutrophils were used for co-inoculation with B16F1 cells to naive mice in the right hind thigh. Tumors (*n* = 9 in each group) were dissected and weighted on d12 after tumor cell inoculation. *p < 0.05, **p < 0.01.

Neutrophils, as a key component in inflammatory response, play a crucial role in inflammation-driven tumorigenesis and tumor progression [26–31]. Intriguingly, the circulating B16F0 cells could promote neutrophil infiltration in lung, and this effect could also be partially impaired by IL-37 (Figure 3A). Moreover, after intravenous inoculation of B16F1 cells, the circulating B16F0 cells could further increase the infiltration of neutrophils in lung (Figure 3B). We then depleted neutrophils *in vivo* (Supplementary Figure 3) to decrease neutrophil infiltration in lung (Figure 3B). In this situation, CTCs were unable to promote tumor metastasis (Figure 3C), suggesting that CTCs could not promote tumor metastasis in the absence of neutrophils, even if CTCs could induce systemic inflammation.

We then further analyzed the effect of CTCs on neutrophil function. For this purpose, mice only received i.v. injection of B16F0 cells, thus preparing the mice with circulating tumor cells. The function of neutrophils could be altered in bone marrow, and the altered function could be maintained after the process of chemotaxis [32]. We therefore isolated neutrophils from the bone marrow and peritoneal cavity of naive mice, B16F1-bearing mice, and circulating B16F0-bearing mice (cir-B16F0-mice), respectively. In co-inoculation test, tumor growth was suppressed by the neutrophils from naive mice. However, the neutrophils from B16F1-bearing mice and cir-B16F0-mice could significantly promote the growth of tumor (Figure 3D). The conversion of neutrophil function in cir-B16F0-mice indicated that the function of neutrophils could be converted in the presence of circulating tumor cells. Moreover, CTCs failed to induce the tumor-promoting function of neutrophils in B16F1-bearing mice and cir-B16F0-mice, if IL-37 was expressed *in vivo* (Figure 3D). Taken together, the above results demonstrated that CTC-induced systemic inflammation could not only promote the infiltration of neutrophils in the target tissues, but also convert neutrophil function to promote tumor growth and metastasis.

### CTCs promote the expression of tumor-promoting genes in neutrophils

**Figure 4:**
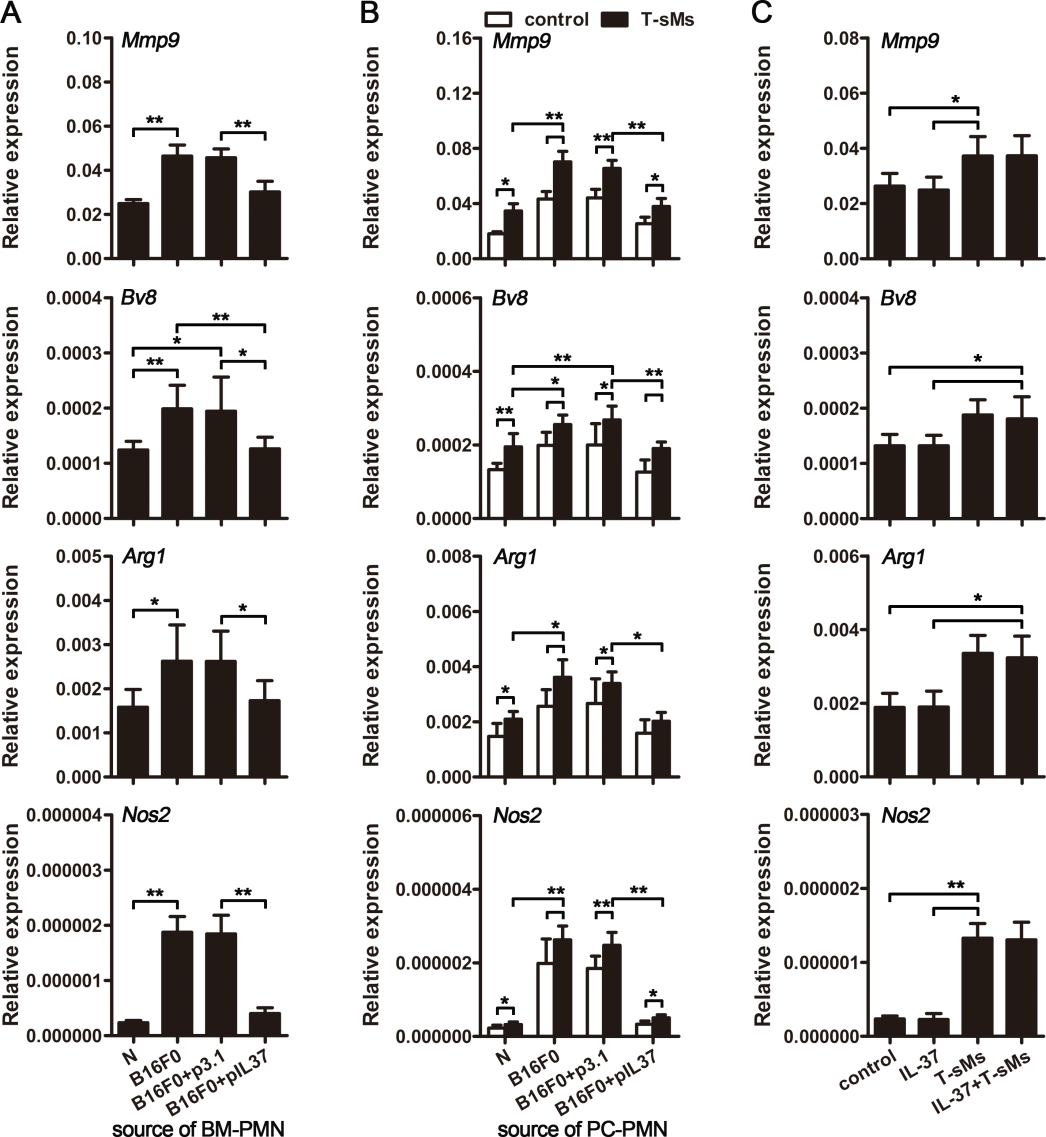
CTCs induce the pro-tumor function of neutrophils (**A** and **B**) Mice (*n* = 9 in each group) were intravenously inoculated with B16F0 cells and treated by i.v. injection of pIL37 plasmid. Neutrophils were isolated from the bone marrow (BM) of mice without further stimulation (A), or isolated from the peritoneal cavity (PC) of mice and stimulated with T-sMs (0.5 mg/ml) for 12 h (B). Naive mice (N) were used as control. The expression of *Mmp9*, *Bv8*, *Arg1* and *Nos2* genes was detected by real-time RT-PCR. (**C**) Neutrophils were isolated from the BM of naive mice, and stimulated with IL-37 (200 ng/ml) and/or T-sMs (0.5 mg/ml) for 12 h. The expression of *Mmp9*, *Bv8*, *Arg1* and *Nos2* genes was detected by real-time RT-PCR. Data are pooled from three independent experiments with a total of six samples in each group (C). *p < 0.05, **p < 0.01.

In order to further explore the effect of CTCs on the protumor function of neutrophils, we analyzed the gene expression of neutrophils from cir-B16F0-mice with or without *in vivo* expression of IL-37. Compared with the neutrophils from naive mice, the neutrophils from cir-B16F0-mice expressed higher levels of genes that are related to tumor-promoting function of neutrophils, including *Mmp9*, *Bv8*, *Arg1* and *Nos2* (Figure 4A and Supplementary Figure 4), suggesting that CTCs could alter the expression of these genes to augment the tumor-promoting function of neutrophils.

We then investigated whether CTCs could alter the response of neutrophils to the stimuli in tumor microenvironment by stimulating neutrophils with soluble molecules from tumor (T-sMs), which might represent complex stimuli in tumor milieu [32]. In the presence of T-sMs, the expression of *Mmp9*, *Bv8*, *Arg1* and *Nos2* was further increased (Figure 4B). Compared to the neutrophils from naive mice, the neutrophils from cir-B16F0-mice showed much higher expression of *Mmp9*, *Bv8*, *Arg1* and *Nos2* (Figure 4B). However, the up-regulation of gene expression and the increase of neutrophil responsiveness to T-sMs were abrogated by *in vivo* expression of IL-37 in cir-B16F0-mice (Figure 4A and 4B). In addition, IL-37 could not directly affects the gene expression of neutrophils from naive mice, when they were stimulated with or without T-sMs (Figure 4C), suggesting that IL-37 could suppress CTC-induced inflammatory response, and the overall inhibition of inflammation affects the gene expression and responsiveness of neutrophils in our models.

### CTCs suppress TRAIL expression and degranulation of neutrophils

**Figure 5:**
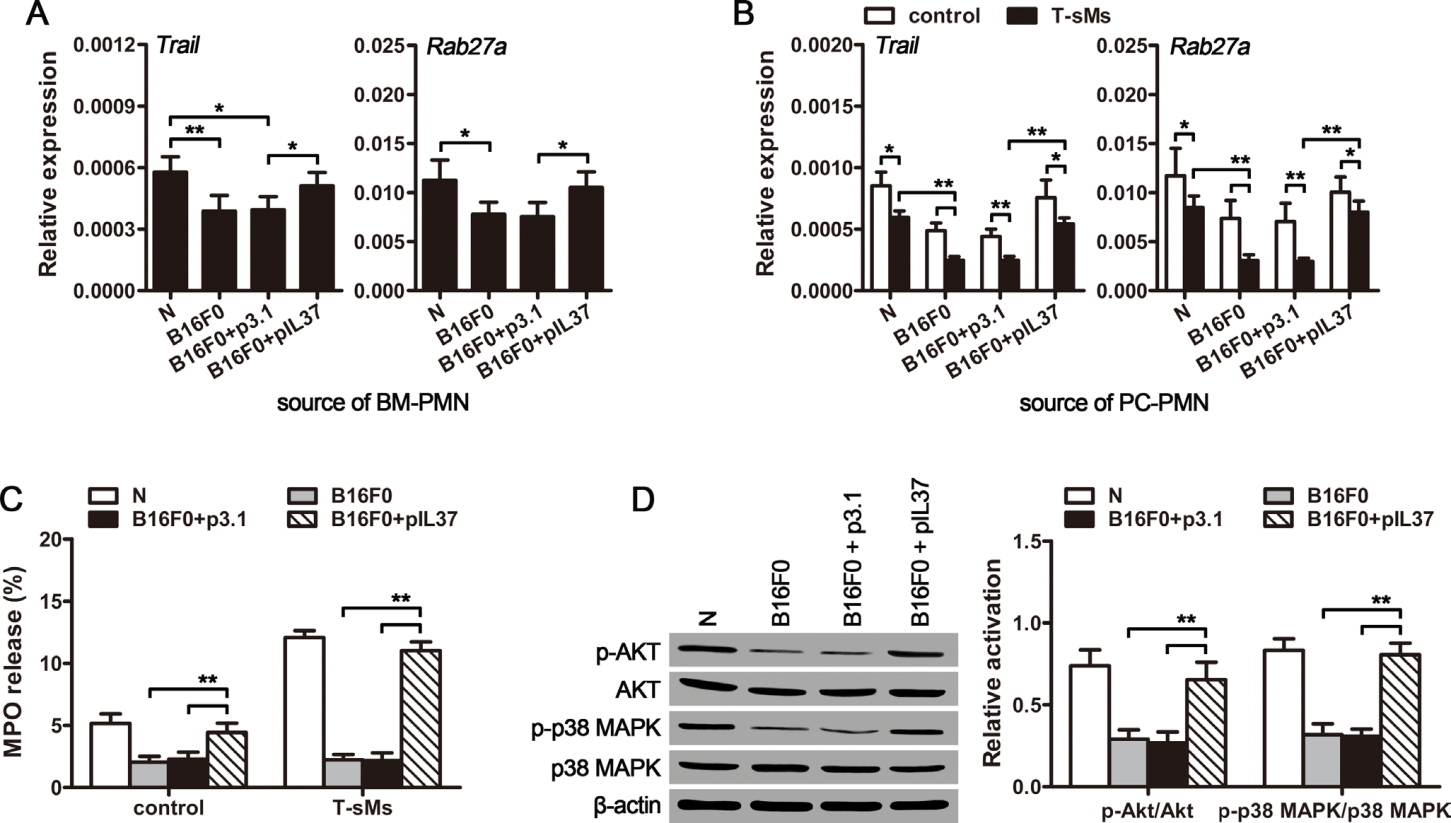
CTCs suppress the anti-tumor function of neutrophils Neutrophils were isolated from the bone marrow (BM) (**A**) or peritoneal cavity (PC) (**B**–**D**) of cir-B16F0-mice (*n* = 8 in each group) with or without treatment with pIL37 plasmid, and stimulated with T-sMs (0.5 mg/ml) for 12 h (B) or 30 min (C and D). The mRNA level of *Trail* and *Rab27a* was detected by real-time RT-PCR (A and B). The release of MPO from neutrophils was detected after stimulation with T-sMs, as described in Methods (C). The phosphorylation of Akt and p38 MAPK was detected by Western blot after stimulation with T-sMs (D, left). The ratios of phospho-Akt to Akt (p-Akt/Akt) and phospho-p38 MAPK to p38 MAPK (p-p38 MAPK/p38 MAPK) were calculated after densitometric analysis of Western blots (D, right). Naive mice (N) were used as control. *p < 0.05, **p < 0.01.

We then investigated the effect of CTCs on the antitumor function of neutrophils. Neutrophils could suppress tumor growth by releasing TRAIL and myeloperoxidase (MPO) in response to suitable stimuli [32–34]. We therefore analyzed TRAIL expression and degranulation (releasing MPO) of neutrophils from cir-B16F0-mice. The results showed that circulating B16F0 cells could decrease the expression of TRAIL at both mRNA level and protein level (Figure 5A and Supplementary Figure 5A). Besides, the expression of Rab27a, which is required for efficient degranulation of neutrophils [35], was also decreased in cir-B16F0-mice (Figure 5A and Supplementary Figure 5A). Furthermore, the expression of *Trail* and *Rab27a* was further reduced in neutrophils, when they were stimulated by T-sMs (Figure 5B). Compared with neutrophils from naive mice, the neutrophils from cir-B16F0-mice showed much lower expression of *Trail* and *Rab27a* (Figure 5B). Consistent with the decreased of Rab27a, the spontaneous and T-sM-induced degranulation of neutrophils from cir-B16F0-mice were impaired, as evaluated by the release of MPO (Figure 5C) and the expression of CD63, a marker related to degranulation [36], on the surface of cells (Supplementary Figure 5B).

To get further insights into the attenuation of neutrophil degranulation in cir-B16F0-mice, we next analyzed the activation of p38 MAPK and PI3K pathways that are required for the induced degranulation of neutrophils [37]. For this purpose, we stimulated neutrophils with T-sMs, which could activate p38 MAPK and PI3K pathways in neutrophils [32]. Intriguingly, the phosphorylation levels of p38 MAPK and Akt in neutrophils from cir-B16F0-mice were significantly lower than those in neutrophils from naive mice (Figure 5D). However, the effect of circulating B16F0 cells on TRAIL expression (Figure 5A and Supplementary Figure 5A) and degranulation (Figure 5C and Supplementary Figure 5B) of neutrophils was also inhibited by *in vivo* expression of IL-37, suggesting that CTC-induced inflammation contributed to the down-regulation of TRAIL expression and the attenuation of neutrophil degranulation.

### CTCs induce inflammatory response by releasing TLR2/4 ligands

**Figure 6:**
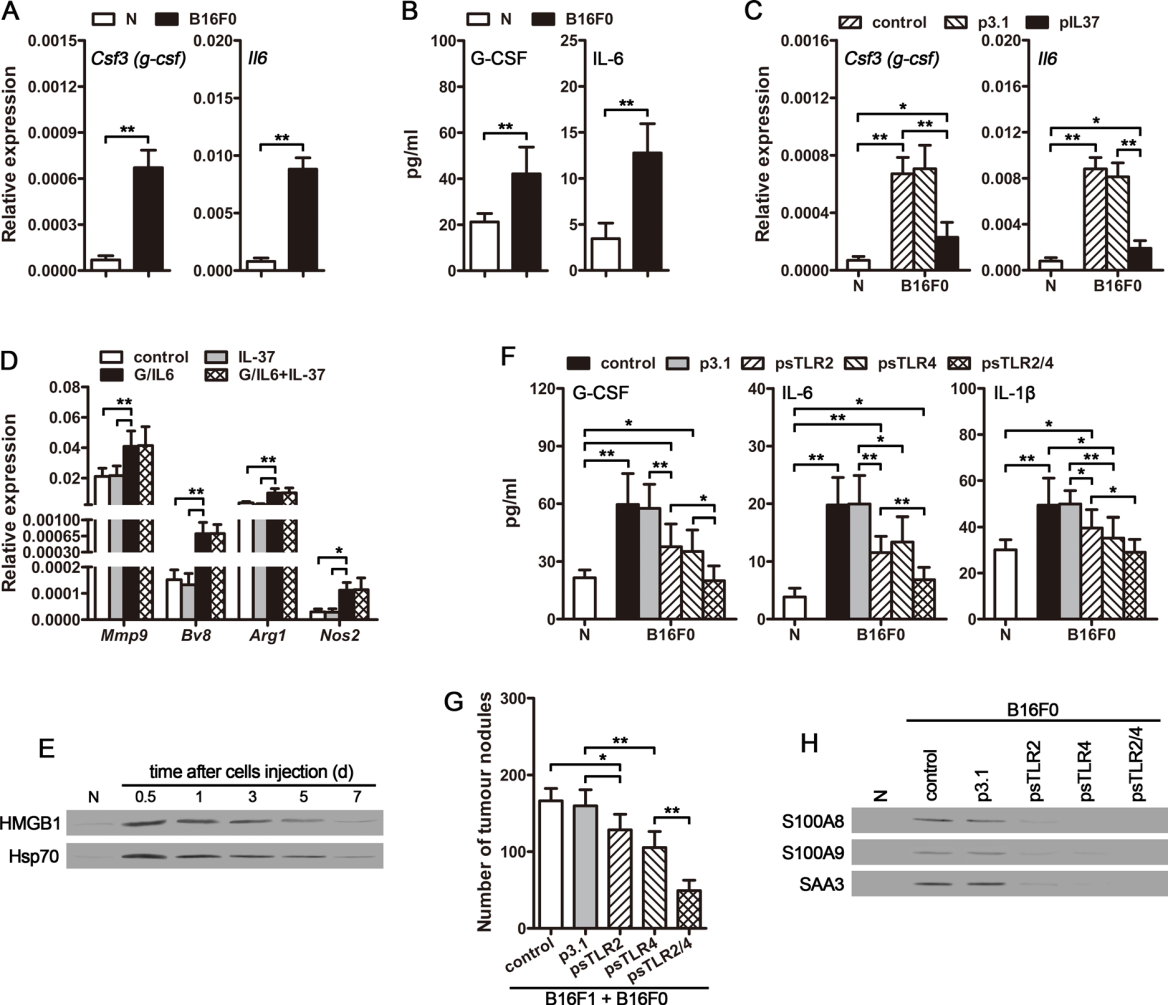
CTCs promote the expression of G-CSF and IL-6 *in vivo* (**A**) The expression of *Csf3 (g-csf)* and *Il6* genes in lung tissues of cir-B16F0-mice was detected by real-time RT-PCR on d4 after primary inoculation. (**B**) Serum levels of G-CSF and IL-6 in cir-B16F0-mice were detected by ELISA on d4 after primary inoculation. (**C**) Cir-B16F0-mice were untreated or treated by i.v. injection of pIL37 plasmid. The expression of *Csf3* (*g-csf*) and *Il6* genes in lung tissues was detected by real-time RT-PCR on d10 after primary inoculation. (**D**) Neutrophils were isolated from the bone marrow of naive mice and stimulated with G-CSF/IL-6 (50 ng/ml of each) and/or IL-37 (200 ng/ml) for 12 h. The expression of *Mmp9*, *Bv8*, *Arg1* and *Nos2* genes was detected by real-time RT-PCR. (**E**) Mice received i.v. injection of B16F0 cells (3 × 10^5^). HMGB1 and HSP70 in the serum of mice were detected by Western blot at the indicated time points. (**F**) Cir-B16F0-mice were untreated or treated by i.v. injection of psTLR2 and/or psTLR4 plasmids. Serum levels of G-CSF, IL-6, and IL-1β were detected by ELISA on d10 after primary inoculation. (**G**) Mice received the i.v. injection of B16F1 cells with B16F0 cells. The mice were untreated or treated by i.v. injection of psTLR2 and/or psTLR4 plasmids. Metastatic nodules on the surface of lung were counted on d12 after B16F1 cell inoculation. (**H**) Cir-B16F0-mice were untreated or treated by i.v. injection of psTLR2 and/or psTLR4 plasmids. S100A8, S100A9 and SAA3 proteins in lung tissues were detected by Western blot on d10 after primary inoculation. Data are representative of three independent experiments (D, E and H). *n* = 9 in each group (A–C, F and G). Naive mice (N) were used as control (A–C, E, F and H). *p < 0.05, **p < 0.01.

The function of neutrophils could be converted from tumor-suppressing to tumor promoting by G-CSF and IL-6 [32]. We then compared the expression of G-CSF and IL-6 in naive mice and cir-B16F0-mice. The mRNA levels of *Csf3* (*g-csf*) and *Il6* genes in lung (Figure 6A) and spleen (Supplementary Figure 6A) were much higher in cir-B16F0-mice. Consistently, compared to naive mice, the serum levels of G-CSF and IL-6 in cir-B16F0-mice were elevated (Figure 6B and Supplementary Figure 6B), which further demonstrated that the function of neutrophils could be converted to tumor-promoting by circulating tumor cells. The increased expression of *Csf3* (*g-csf*) and *Il6* in cir-B16F0-mice could be significantly suppressed by *in vivo* expression of IL-37 (Figure 6C and Supplementary Figure 6C) or blocking IL-1β *in vivo* (Supplementary Figure 6D and 6E). However, IL-37 could not directly influence the effect of G-CSF/IL-6 on neutrophils (Figure 6D). These results indicated that the increased production of proinflammatory cytokines *in vivo* was crucial for the effect of CTCs.

To explore the inflammatory stimuli from CTCs that stimulate G-CSF and IL-6 expression, we focused on the tumor cell-derived TLR2 and TLR4 (TLR2/4) ligands, since IL-37 could suppress TLR4 ligand-induced inflammatory response [22, 38]. HMGB1 and HSP70, the representatives of TLR2/4 ligands released from tumor cells, have been implicated in promoting the production of proinflammatory cytokines [39, 40]. As shown in Figure 6E, the protein levels of HMGB1 and HSP70 were elevated in the serum of mice, and could be sustained for several days after intravenous injection of B16F0 cells. Intriguingly, the elevated serum levels of G-CSF, IL-6 and IL-1β in cir-B16F0-mice could be dramatically suppressed by the expression of soluble TLR2 (sTLR2) and soluble TLR4 (sTLR4) *in vivo* (Figure 6F). Consistently, the promotion effect of circulating B16F0 cells on the metastatic colonization of disseminated B16F1 cells was also suppressed by sTLR2 and sTLR4 (Figure 6G), further confirming that TLR2/4 ligands are the main inflammatory stimuli that are responsible for promoting tumor metastasis by CTCs.

Moreover, the expression of S100A8, S100A9 and SAA3, which are also the ligands for TLR2/4 and have been implicated in promoting G-CSF, IL-6 and IL-1β expression [41–4], in lung tissues were increased in cir-B16F0-mice (Figure 6H). The increased expression of these ligands was also suppressed by *in vivo* expression of sTLR2 and sTLR4 (Figure 6H), suggesting that CTC-derived TLR2/4 ligands could induce more endogenous TLR2/4 ligands expression in tissue, thus efficiently inducing systemic inflammation and promoting the metastatic colonization of disseminated carcinoma cells.

## DISCUSSION

The metastatic colonization of disseminated carcinoma cells in distant organ is the most critical and rate-limiting step of metastasis, which is influenced by different factors. Inflammation has been recognized as a key player in cancer development, which can fuel both primary tumor growth and metastasis [10, 11, 21]. Our results confirmed that inflammation is indeed essential for the metastatic colonization. In our models, the inoculation with metastatic B16F1 cells could trigger an inflammatory response and induce the conversion of neutrophil function, which might be due to the existence of metastatic foci in the lung, or the B16F1 cells that did not extravasate, and retained as CTCs. By employing non-metastatic B16F0 cells, we confirmed that CTCs could trigger the systemic inflammation, which induced the conversion of neutrophil function, promoted neutrophil infiltration in the lung, and increased the expression of pro-metastatic proteins such as MMP-9, Bv8, S100A8, and S100A9 [13] in the lung. Moreover, the CTC-induced inflammation is the main mechanism underlying the promoting effect of CTCs on the metastatic colonization of disseminated carcinoma cells, since inhibiting the inflammatory response with IL-37 could efficiently suppress the effect of CTCs. Therefore, the anti-inflammatory therapy might be very important when the higher CTC count is detected.

Neutrophils are functionally plastic in the tumor microenvironment. They have been proposed to inhibit metastatic seeding in the lung by generating H_2_O_2_ [45]. In contrast, they also have been reported to be account for metastasis in the lung by supporting lung colonization of metastasis-initiating breast cancer cells [31]. Our data showed that neutrophils are required for CTCs to promote tumor metastasis. CTCs could suppress the anti-tumor function of neutrophils by down-regulating the expression of TRAIL and attenuating the degranulation of neutrophils. On the other hand, CTCs could up-regulate the expression of tumor-promoting genes, including *Mmp9*, *Bv8*, *Arg1* and *Nos2*. Therefore, the functional phenotype of neutrophils could be converted by CTCs from tumor-suppressing to tumor-promoting. The tumor-promoting neutrophils are required for the initiation of angiogenesis in metastatic lesions [46], which is crucial for the formation of metastases. Thus, the conversion of neutrophil function was crucial for CTCs to promote tumor metastasis. Consistently, our data showed that depleting neutrophils *in vivo* could abrogate the promoting effect of CTCs on the metastatic colonization.

Neutrophils can be polarized into either an antitumoral (N1) or a protumoral (N2) phenotype, which depends on external cues in the environment [18, 32]. Our results showed that the conversion of neutrophil function was due to CTC-induced systemic inflammation, since inhibiting CTC-induced inflammatory response with IL-37 could suppress CTC-induced conversion of neutrophil function. More specifically, the CTC-induced production of G-CSF and IL-6 was accounted for CTC-induced neutrophil function alteration.

CTCs could be an important source of inflammatory stimuli, especially the ligands for TLR2 and TLR4 (TLR2/4) that have been known to induce the production of proinflammatory cytokines, such as G-CSF, IL-6, IL-1β, TNF-α [41, 44, 47]. After intravasation, most of tumor cells in circulation are damaged by the shear stress and mechanical stresses in blood, thus releasing intracellular molecules, many of which have been identified as the ligands for TLR2/4, including HMGB1, HSP60, HSP70, gp96, S100A8, S100A9, SAA3 and so on [48]. The half-life of the CTCs in blood of patients is only 1 to 2 hours [49]. The CTCs that are dying are replenished quickly, relying on the continuous entry of tumor cells into blood [49]. The continuous entry of tumor cells into blood and the rapid death of CTCs suggest the continuously release of TLR2/4 ligands into blood. CTC-released TLR2/4 ligands could induce the production of IL-1β, which has been implicated in promoting the expression of S100A8, S100A9 and SAA3 [49]. As shown in our data, CTC-released TLR2/4 ligands could further induce the production of S100A8, S100A9 and SAA3 that are also the ligands for TLR2/4 and have been reported to promote the production of G-CSF, IL-6 and IL-1β [41–44]. Thus, CTC-derived TLR2/4 signal could be amplified *in vivo*, and eventually efficient in inducing the expression of G-CSF and IL-6 *in vivo*.

In sum, we identify a role of CTCs in promoting tumor metastasis by inducing a systemic inflammatory response. The increased CTCs could promote neutrophil infiltration, induce the polarization of neutrophils to a pro-metastasis phenotype, and thus facilitate the metastatic colonization of disseminated carcinoma cells in distant organs. Therefore, the higher counts of CTCs could increase the risk of metastasis by inducing systemic inflammation, resulting in the poorer prognosis and shorter survival of the patients. On the other hand, our study also confirmed that the promoting effect of CTCs on tumor metastasis could be abrogated by either inhibiting the inflammatory response or blocking TLR2/4 signaling. Although the “N2” phenotype neutrophils are crucial for the promoting effect of CTCs on tumor metastasis, the protracted depletion of neutrophils is clinically untenable. Thus, inhibiting inflammatory response to reduce the production of cytokines such as G-CSF and IL-6, or blocking the ligands for TLR2/4, when the increased CTC counts is detected, might be very important for designing the therapeutic strategy to prevent tumor metastasis. More research on the metastatic axis of CTCs, tumor-associated systemic inflammation, neutrophil recruitment and function alteration may reveal effective targets to prevent and treat tumor metastasis.

## MATERIALS AND METHODS

### Animals and cell lines

BALB/c mice, 6-8 weeks old, were purchased from Center of Medical Experimental Animals of Hubei Province (Wuhan, China) for studies. All animal experiments were approved by the Animal Care and Use Committee of Tongji Medical College. C57BL/6 background B16F0 and B16F1 melanoma cells were purchased from China Center for Type Culture Collection (CCTCC, Wuhan, China) and cultured according to their guidelines.

### Reagents and plasmids

Murine G-CSF and IL-6 were purchased from PeproTech (Rocky Hill, NJ). Recombinant human IL-37 was purchased from R&D Systems. Anti-Ly6G mAb was purchased from BioExpress (clone 1A8). Plasmids pIL37 and pCXCL1 are expression vectors carrying the cDNA encoding human IL-37 (IL-37b) and murine CXCL1, respectively. Plasmids psTLR2 and psTLR4 are expression vectors carrying the cDNA encoding the extracellular domain of murine Toll-like receptor 2 and 4, respectively. These plasmids were constructed by the insertion of cDNA into the mammalian expression vector pcDNA3.1 (Invitrogen, Carlsbad, CA) in our laboratory. All of the vectors were identified by *in vivo* expression (Supplementary Figure 7).

### Assay of tumor cell arrest in lung and extravasation

Carboxyfluorescein succinimidyl ester (CFSE)-labeled B16F0 and B16F1 cells (5 × 10^5^ cells/mice) were injected intravenously to mice. Lungs were harvested 5 and 24 h after tumor cell injection. Frozen sections were prepared and analyzed by fluorescence microscopy. Fluorescent spots were counted from randomly chosen fields in the sections of each mouse.

### Animal experiments and treatment protocol

In tumor cell metastasis model, mice received i.v. injection of 3 × 10^5^ B16F1 cells on d0. When indicated, B16F0 cells (3 × 10^5^) were injected together with B16F1 cells, followed by another i.v. injection of B16F0 cells (3 × 10^5^) on d6. The tumor nodules on the surface of lung were counted on d12 after inoculation of B16F1 cells.

To prepare the mice with circulating tumor cells, mice received i.v. injection of B16F0 cell (3 × 10^5^) on d0 and d6 respectively. The mice were designated as cir-B16F0-mice, and used for other experiments on d12 after the first injection of B16F0 cells. When indicated, cir-B16F0-mice received i.v. injection of the indicated plasmid DNA (100 μg of each per injection), once every 2 days starting from d1 after primary inoculation. PBS and pcDNA3.1 (p3.1) were used as controls.

To analyze the effect of IL-37 on neutrophils *in vivo*, B16F1-bearing mice or cir-B16F0-mice received i.v. injection of pIL-37 plasmid (100 μg of each per injection) from d1 to d11, once every 2 days. Plasmid p3.1 was used as control. The mice were used for isolation of neutrophils on d12 after the first injection of tumor cells.

In co-inoculation test, mice received intramuscular injection of B16F1 cells (2 × 10^5^) in the right hind thigh. When indicated, B16F1 cells were mixed with 1 × 10^6^ neutrophils, which were isolated from naive mice (N) or B16F1-bearing mice or cir-B16F0-mice with or without the treatment by pIL-37 plasmid. Tumors were dissected and weighed on d12 after tumor cell inoculation.

### *In vivo* gene transfection

Plasmids were prepared and analyzed as described previously [51]. Mice received the injection of plasmid DNA (100 μg) via the tail vein (i.v. injection) using the hydrodynamics-based gene delivery technique [51].

### *In vivo* depletion of neutrophils

To deplete neutrophils, anti-Ly6G antibody (clone 1A8, BioExpress) was used [52]. Mice received i.p. injection of anti-Ly6G antibody at a dose of 300 μg in 500 μl PBS on -d1, d1, d4, d7, d10 of tumor inoculation [53]. The depletion of neutrophils was identified by flow cytometric analysis (Supplementary Figure 3).

### Recruitment of neutrophils to peritoneal cavity

To recruit neutrophils to peritoneal cavity, CXCL1-expressing hepatocytes were injected to peritoneal cavity of mice as described previously [32]. 12 h later, the peritoneal cells were harvested for the isolation of neutrophils.

### Isolation of neutrophils

Murine neutrophils were isolated from bone marrow cells, peripheral blood cells, or peritoneal cells as described previously [54]. Briefly, the cells were washed once in HBSS, layered over a Percoll gradient (54%/64%/72% for bone marrow cells, 54%/66%/78% for peripheral blood cells, and 54%/64%/80% for peritoneal cells), and centrifuged at 1060 × *g* for 30 min. The dense bands at 64%/72%, 66%/78%, or 64%/80% interface were collected as neutrophil fraction. The isolated cells were 90% neutrophils as assessed by flow cytometric analysis (Supplementary Figure 8).

### Preparation of soluble molecules from tissues

Palpable tumors were dissected. The mixture of soluble molecules from tumor (T-sMs) was prepared by digesting the tissues with collagenase and removing debris by centrifugation. The concentration of T-sMs was defined by the concentration of protein, which was determined by using Coomassie Bradford reagent (Thermo Fisher Scientific, Rockford, IL) according to the manufacturer's instructions.

### Other methods

Other methods were performed using standard protocols, including soft agar assay, cell proliferation assay, analysis of gene expression by conventional RT-PCR and real-time RT-PCR, Western blot assay, assay of degranulation, immunohistochemistry, cytokine neutralization, ELISA analysis, flow cytometric analysis. See Supplementary Methods for details.

### Statistical analysis

Results were expressed as mean value ± SD and interpreted by one-way ANOVA. Differences were considered statistically significant when *P* <0.05.

## SUPPLEMENTARY MATERIALS FIGURES AND TABLES


