# Supplementary material for: Circulating tumor cells promote the metastatic colonization of disseminated carcinoma cells by inducing systemic inflammation
Source: Oncotarget. 2017 Mar 10;8(17):28418–30. doi: 10.18632/oncotarget.16084 (PMC5438660; doi:10.18632/oncotarget.16084)
Supplement: Supplementary file 1 [file oncotarget-08-28418-s001.pdf]

Circulating tumor cells promote the metastatic colonization of disseminated carcinoma cells by inducing systemic inflammation

Supplementary Materials

SUPPLEMENTARY MATERIALS AND METHODS

Soft agar assay

B16F1 cells were harvested and resuspended in 0.33% agar in DMEM (20% FBS) and plated (2 × 10^3 cells/well in 6-well plates) on a layer of 0.6% agar in DMEM (20% FBS) in triplicate. After 3 weeks culture in the presence or absence of IL-37, the colonies of tumor cells were photographed and counted under a microscope. These experiments were independently repeated at least 3 times. Colonies with more than 50 cells were counted.

Cell proliferation assay

Cell proliferation was measured with the Cell Counting Kit-8 (CCK-8) assay kit (Dojindo Corp.). 1 × 10^3 B16F1 cells were plated into each well of a 96-well plate, and pre-incubated in the presence or absence of IL-37 (200 ng/ml) for 24 h. Then the cells were washed twice, and 90 µl fresh medium and 10 µl CCK-8 were added. The cells were subsequently incubated for 2 h at 37°C and the attenuance was measured at 450 nm. Three independent experiments were performed.

Analysis of gene expression by conventional RT-PCR and real-time RT-PCR

Total RNA was extracted from neutrophils with TRIzol reagent (Invitrogen) or from tissues (lung, spleen) homogenized in TRIzol according to the manufacturer's instructions. The relative quantity of mRNA was determined by real-time RT-PCR according to MIQE guideline [55]. *Actb* was chosen as reference gene. The relative expression of gene was calculated using GeNorm software. The primer sequences were as follows: *Mmp9*, sense 5′-TGCCCAGCGACCACA ACTC-3′, antisense 5′-CGGACCCGAAGCGGACATT-3′; *Bv8*, sense 5′-TGCTACTTCTG CTGCTACC-3′, antisense 5′-CCGCACTGAGAGTCCTTGTC-3′; *Trail*, sense 5′’-TACTGGGATCACTCGGAGAAG-3′, antisense 5′-ACGTGGTTGAGAAAT GAATGCC-3′; *Rab27a*, sense 5′-CGTGCCTTCCAGCGTTGTTC-3′, antisense 5′-TCGCCT GCCTCTGCTTCTCA-3′; *Arginase1* (*Arg1*), sense 5’-TGGGAAGACAGCAGAGGA-3′, antisense 5′-TCAGTCCCTGGCTTATGG-3′; *Nos2*, sense 5′-GGAGCGAGTTGTGGATT GTC-3′, antisense 5′-TGA GGGCTTGGCTGAGTGA-3′; *Csf3* (*g-csf*), sense 5′-AGGGAAG GAGATGGGTAAAT-3′, antisense 5′-CGGAAGGGAGACCAGATGC-3′; *Il6*, sense 5′-GC TGGAGTCACAGAAGG AG-3′, antisense 5′-TAGGTTTGCCGAGTAGA-3′; *Actb* (*β-actin*), sense 5′-AGGGAAATCGTGCGTGAC-3′, antisense 5′-CGCTCGTTGCCAATAGTG-3′.

For conventional RT-PCR assays, the primer sequences were as follows: *Il18r*, sense 5′-TTAGGACCAAAGTGTGAGAAGG-3′, antisense 5′-TCTCGTCTCTTTCCGCTATGCG-3′; *Sigirr* (*Il1r8*), sense 5′-TGGAGATGAACGATGGGAAGT-3′, antisense 5′-AAGATAGGTCT GCGGGTG-3′; human *IL-37b*, sense 5′-CAGCCTCTGCGGAGAAAGGAAGT-3′, antisense 5′-GTTTCTCCTTCTTCAGCTGAAGGGATGGAT-3′; *Tlr2*, sense 5′-TTTCACCACTGCCC GTAGAT-3′, antisense 5′-TCGCTCACTACGTCTGACTC-3′; mouse *Tlr4*, sense 5′-CCCTC AGCACTCTTGATTGC- 3′, antisense 5′-TGCTTCTGTTCCTTGACCCA-3′; *Actb* (*β-actin*), sense 5′-ATCTCCTGCTCGAAGTCTAGAG-3′, antisense 5′-ATGGGTCAGAAGGACTCC TATG-3′.

Western blot assay

Western blot assay was accomplished as described previously [56]. Abs were purchased from Cell Signaling Technology (Beverly, MA) and Santa Cruz Biotechnology (Santa Cruz, CA).

Assay of degranulation

To induce the degranulation, neutrophils were stimulated for 30 min with T-sMs (0.5 mg/ml). The release of azurophilic granules was determined by detecting the release of MPO. Percentage of MPO release was calculated as described previously [32]. Meanwhile, CD63, which is associated with the primary granules and transferred to the surface of neutrophils during degranulation [36], was detected by flow cytometry with PE-conjugated anti-mouse CD63 (BD Biosciences, San Diego, CA).

Immunohistochemistry

Tissue sections were prepared and subjected to immunohistochemical analysis, as described earlier [51]. Anti-mouse Ly6G Ab (Santa Cruz Biotechnology) was used as primary Abs for detecting neutrophils. HRP-conjugated Goat anti-Rabbit IgG was used as the secondary antibody. Images were obtained using an Olympus-IX71 microscope at 40 × 10 magnification. The neutrophils were counted using Image-Pro Plus 6.0 software. The neutrophil density was defined as the number of neutrophils per microscopic field.

Cytokine neutralization

For IL-1β neutralization experiments, mice were injected intraperitoneally with 50 μg anti-IL-1β twice weekly (clone B122; BioXCell). Control mice received equal amounts of isotype control antibody or equal volume of PBS.

ELISA analysis

Serum levels of G-CSF, IL-6 and IL-1β were detected using mouse G-CSF, IL-6 and IL-1β ELISA kits (R&D Systems, Minneapolis, MN), according to manufacturer′s instruction.

Flow cytometric analysis

PE-Cy7-conjugated anti-mouse CD11b, PE-conjugated anti-mouse Ly6G, PE-conjugated anti-mouse Ly6C, PE-conjugated anti-mouse Gr-1, and PE-conjugated anti-mouse F4/80 were purchased from eBioscience (San Diego, CA).

To analyze the effect of neutrophil depletion *in vivo*, mice were sacrificed one day after the second injection of antibody. The heparinized blood was harvested for analysis. 20 μl of whole blood was incubated with PE-Cy7-anti-CD11b and PE-anti-Ly6G for 30 min on ice. RBCs were then lysed. The samples were centrifuged at 350 × g for 5 min, and then resuspended in 300 μl of PBS for flow cytometric analysis. $CD11b^{+}Ly6G^{+}$ cells were considered as neutrophils. For controlling the depletion of neutrophils, blood white cells were also analyzed by staining with PE-anti-Gr-1 and PE-anti-Ly6C.

When neutrophils were isolated using Percoll gradient, the isolated cells were assessed by flow cytometric analysis using PE-Cy7-anti-CD11b and PE-anti-Ly6G or PE-anti-Ly6C antibodies (eBioscience). The staining with PE-anti-F4/80 was used for identifying monocytes/macrophages.

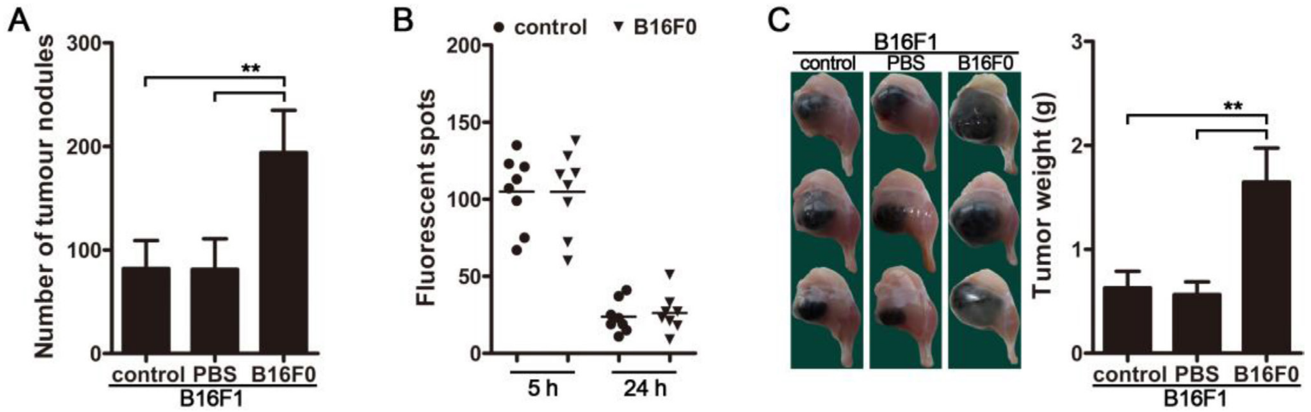

Supplementary Figure 1: CTCs promote tumor growth. (**A**) Mice ($n = 9$ in each group) received i.v. injection of 3×10^5 B16F1 cells on d0, or mice received i.v. injection of 3×10^5 B16F1 cells and 1×10^3 B16F0 cells on d0, followed by i.v. injection of B16F0 cells (1×10^3) on d2, d4, d6, d8 and d10. PBS was used as control. The tumor nodules on the surface of lung were counted on d12 after inoculation of B16F1 cells. (**B**) Mice were injected with CFSE-labeled B16F1 cells (5×10^5) 12 h after i.v. injection of non-labeled B16F0 cells (1×10^3). The mice ($n = 8$ in each group) were sacrificed 5 h or 24 h after B16F1 cell injection. Tumor cells in frozen sections were counted. (**C**) Mice ($n = 9$) were intramuscularly inoculated with B16F1 cells in the right hind thigh, with or without i.v. injection of B16F0 cells, as described in Methods. PBS was used as control. Tumors (left) were dissected and weighted (right) on d12 after the inoculation of B16F1. $^{**}p < 0.01$.

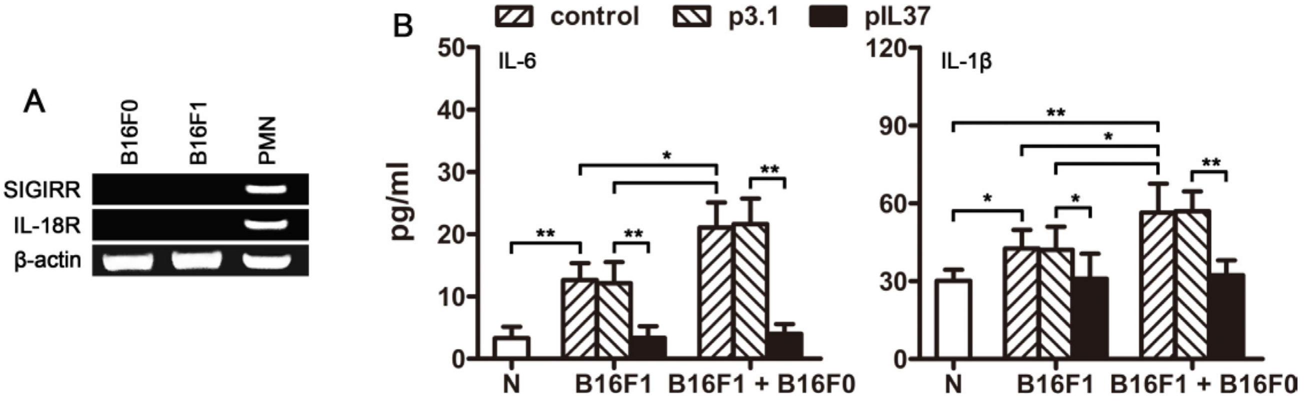

Supplementary Figure 2: CTCs induce the production of pro-inflammatory cytokine. (**A**) The gene expression of IL-37 receptor (*Il18r* and *Sigirr* (*Il1r8*)) in B16F0 and B16F1 cells was detected by RT-PCR. Neutrophils (PMN) were used as positive control. (**B**) Mice were intravenously inoculated with B16F1 cells followed by i.v. injection of B16F0 cells, and received pIL37 plasmid treatment. Serum levels of IL-6 and IL-1β were detected by ELISA on d10 after primary inoculation. Data are pooled from three independent experiments with a total of nine samples in each group (B), or representative of three independent experiments (A). *$p < 0.05$, **$p < 0.01$.

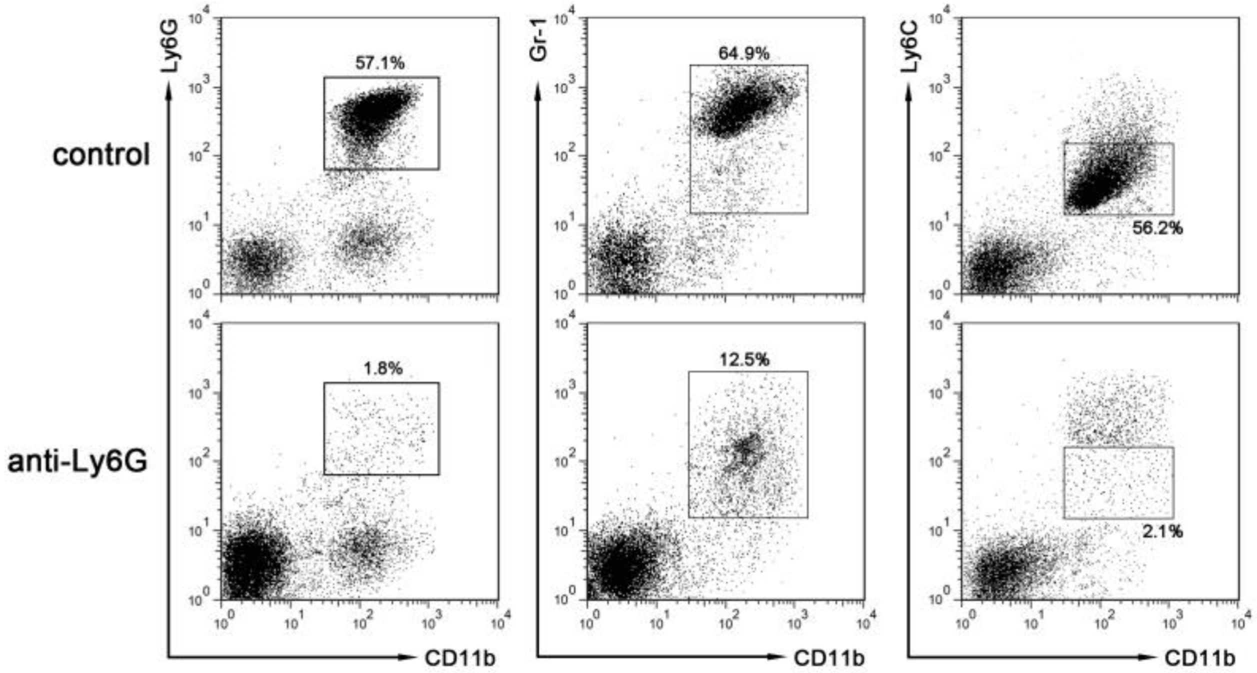

Supplementary Figure 3: Analysis of neutrophil depletion *in vivo*. Neutrophils were depleted *in vivo* as described in Methods. One day after the second injection of the antibody, the neutrophils in blood were detected by flow cytometry as described in Methods. The representative flow cytometry tracings are shown.

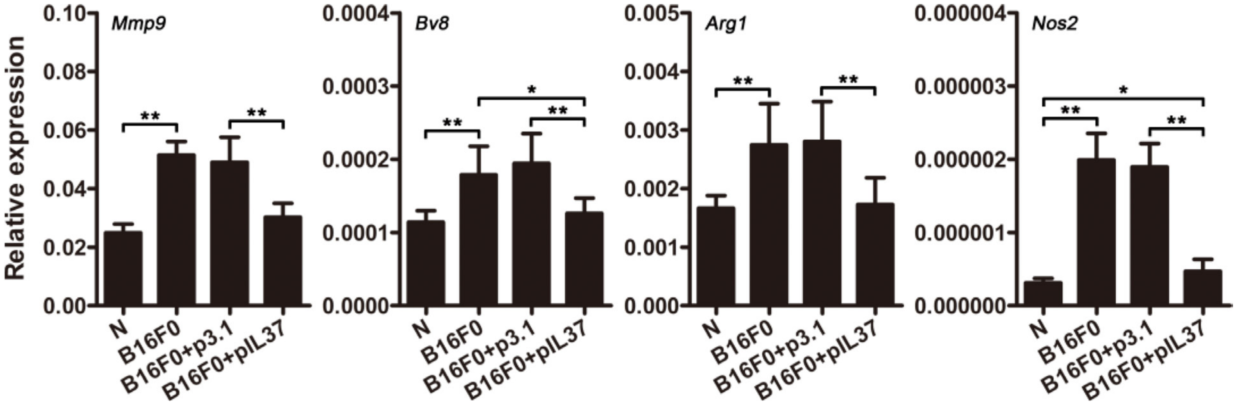

Supplementary Figure 4: CTCs induce the pro-tumor function of neutrophils. Mice (n = 9 in each group) were intravenously inoculated with B16F0 cells and treated by i.v. injection of pIL37 plasmid. Neutrophils were isolated from the peripheral blood of mice as described in Methods. The dense band at 66%/78% interface was collected as neutrophil fraction. Naive mice (N) were used as control. The expression of *Mmp9*, *Bv8*, *Arg1* and *Nos2* genes was detected by real-time RT-PCR. *$p < 0.05$, **$p < 0.01$.

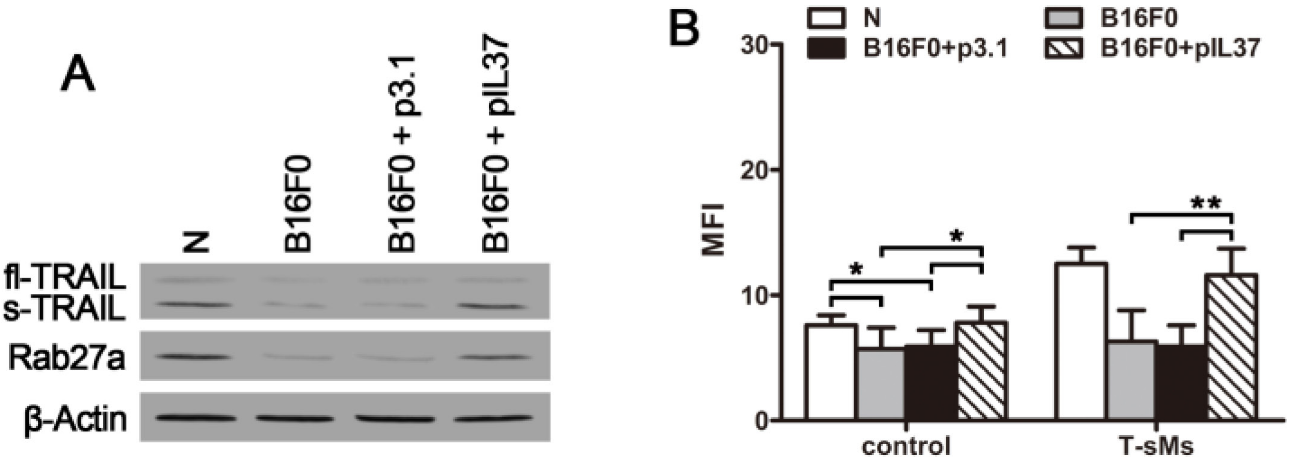

Supplementary Figure 5: CTCs suppress TRAIL and Rab27a expression in neutrophils. (**A**) Neutrophils were isolated from the bone marrow of cir-B16F0-mice, with or without IL-37 expression in vivo. The expression of TRAIL and Rab27a proteins was detected by Western blot. Neutrophils from naive mice (N) were used as control. (**B**) Neutrophils were isolated from the peritoneal cavity of cir-B16F0-mice (n = 8 in each group) with or without treatment with pIL37 plasmid, and stimulated with T-sMs (0.5 mg/ml) for 30 min. The CD63 on the surface of the cells was detected by Flow cytometry. *$p < 0.05$, **$p < 0.01$.

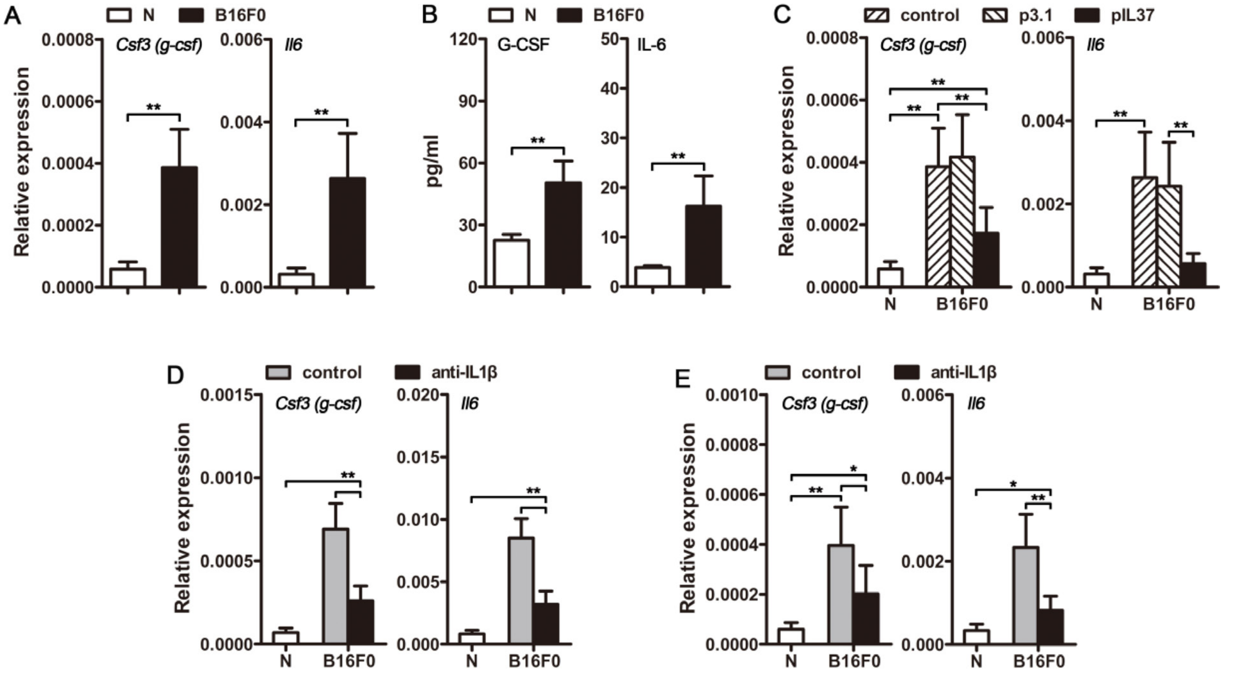

Supplementary Figure 6: CTCs induce the expression of *Csf3 (g-csf)* and *Il6* in spleen. (**A**) The expression of *Csf3 (g-csf)* and *Il-6* genes in the spleen tissues of cir-B16F0-mice ($n = 9$ in each group) was detected by real-time RT-PCR on d4 after first injection of tumor cells. (**B**) Mice ($n = 9$ in each group) received i.v. injection of B16F0 cells (1×10^3), once every 2 days, for 10 days. Serum levels of G-CSF and IL-6 were detected by ELISA on d12 after first injection. (**C**) Cir-B16F0-mice ($n = 9$ in each group) were untreated or treated by i.v. injection of pIL37 plasmid as described in Methods. The expression of *Csf3 (g-csf)* and *Il-6* genes in spleen tissues was detected by real-time RT-PCR on d10 after first injection of tumor cells. (**D** and **E**) Cir-B16F0-mice were untreated or treated with anti-IL-1β antibody to neutralize IL-1β *in vivo*. The expression of *Csf3 (g-csf)* and *Il6* genes in lung tissues (D) and spleen (E) was detected by real-time RT-PCR on d10 after first injection of tumor cells. Naive mice (N) were used as control in each experiment. $*p < 0.05$, $**p < 0.01$.

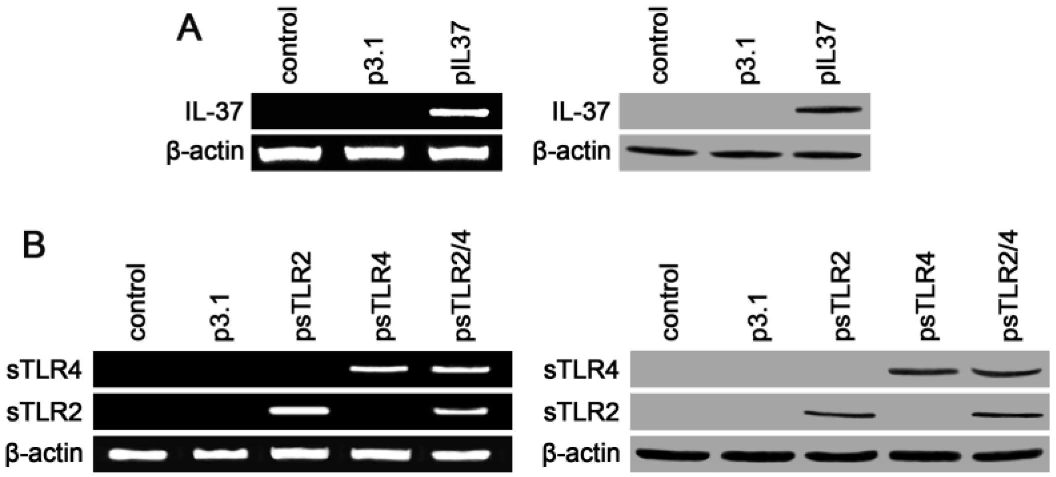

Supplementary Figure 7: Identification of expression vectors by *in vivo* transfection. Mice received i.v. injection of plasmid DNA. pIL37 (**A**), psTLR2 and psTLR4 (**B**) plasmids (100 μg per mouse) were injected alone or injected as a mixture as indicated. Saline (control) and pcDNA3.1 plasmid (p3.1) were used as controls. The livers were surgically excised 48 h or 72 h later, and homogenized. The expression of the vectors was detected at mRNA level by RT-PCR (48 h) and protein level by Western blot (72 h). Anti-human IL-37, anti-mouse TLR2 and anti-mouse TLR4 antibodies were used for Western blot analysis. Data are representative of three independent experiments.

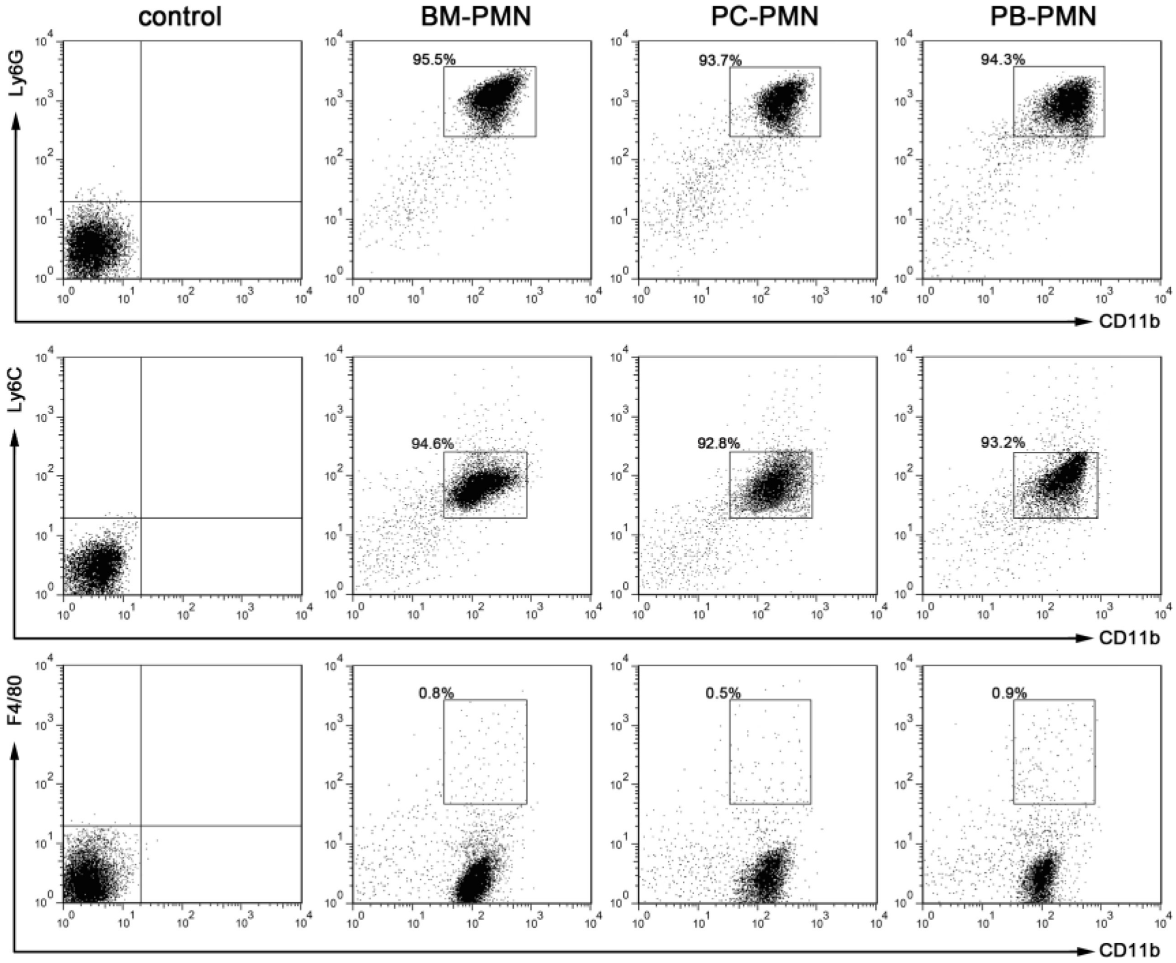

Supplementary Figure 8: Analysis of the isolated neutrophils. Neutrophils were isolated from bone marrow (BM), peritoneal cavity (PC) or peripheral blood (PB) of naive mice as described in Methods. The cells were stained with PE-Cy7-anti-CD11b, PE-anti-Ly6G, and PE-anti-Ly6C, PE-anti-F4/80, and used for flow cytometric analysis. Data are representative of three independent experiments. The representative flow cytometry tracings are shown.
